# Supplementary material for: Serum Zinc and Long-Term Prognosis after Acute Traumatic Brain Injury with Intracranial Injury: A Multicenter Prospective Study
Source: J Clin Med. 2022 Nov 1;11(21):6496. doi: 10.3390/jcm11216496 (PMC9654715; doi:10.3390/jcm11216496)
Supplement: Supplementary file 1 [file jcm-11-06496-s001.zip › jcm-1982437-supplementary.pdf]

Supplementary Table S1. Independent predictive performance of serum zinc levels on long- and short-term neurological prognoses of TBI patients with intra-cranial injury.

Cut-Off Value, Serum Zinc	Accuracy	Sensitivity	Specificity	PPV	NPV
6-month mortality					
80.0mcg/dl	0.7618	0.9271	0.1456	0.8018	0.3488
100.0mcg/dl	0.6489	0.7083	0.4272	0.8218	0.2821
120.0mcg/dl	0.4867	0.4271	0.7087	0.8454	0.2491
6-month disability, GOS 1–3					
80.0mcg/dl	0.6940	0.9300	0.1319	0.7185	0.4419
100.0mcg/dl	0.6099	0.7055	0.3819	0.7311	0.3526
120.0mcg/dl	0.4928	0.4227	0.6597	0.7474	0.3242
1-month mortality					
80.0mcg/dl	0.7782	0.9286	0.1579	0.8198	0.3488
100.0mcg/dl	0.6612	0.7117	0.4526	0.8429	0.2756
120.0mcg/dl	0.4784	0.4235	0.7053	0.8557	0.2287
1-month disability, GOS 1–3					
80.0mcg/dl	0.6858	0.9267	0.1233	0.7117	0.4186
100.0mcg/dl	0.6099	0.7067	0.3836	0.7281	0.3590
120.0mcg/dl	0.4928	0.4223	0.6575	0.7423	0.3276

PPV, positive predictive value; NPV, negative predictive value.
